# Exploring the impact of differences between AI streamers and human streamers on consumer purchase intention in live e-commerce: A grounded theory approach

**DOI:** 10.1371/journal.pone.0334530

**Published:** 2025-10-16

**Authors:** Jie Li, Jinni Chen, Yi Shi, Yi Cui

**Affiliations:** 1 Hangzhou Dianzi University Information Engineering College, Hangzhou, Zhejiang, China; 2 Communication University of China, Beijing, China; Institute for Biocomputation and Physics of Complex Systems, University of Zaragoza, SPAIN

## Abstract

With the rapid development of live e-commerce, AI streamers have gradually emerged as a new industry trend. However, significant differences exist between AI streamers and human streamers in terms of interaction styles, emotional expression, and user experience. How these differences influence consumer purchase intention has not yet been systematically investigated. Based on this, the present study employs grounded theory and the stimulus-organism-response model to explore the mechanism through which the differences between AI streamers and human streamers in live e-commerce affect consumer purchase intention. Data were collected through in-depth interviews and analyzed using open coding, axial coding, and selective coding. A research model was constructed, incorporating sensory differences, affective differences, and functional differences (stimulus factors), perceived entertainment and social presence (organism factor), and purchase intention (response factor), with consumer purpose as a moderating factor. Our results reveal that AI and human streamers differ in sensory, affective, and functional dimensions. Under purchase motivation, AI streamers, with their standardized and concise sensory presentation, better meet the need for quick decision-making, thus outperforming human streamers in functional differences. In contrast, under viewing motivation, human streamers excel in all three dimensions. These differences affect purchase intention through the mediating roles of perceived entertainment and social presence. Consumer purpose (purchase vs. viewing) moderates these effects. This study bridges the gap in understanding how AI and human streamers’ differences shape consumer behavior, offering practical insights for e-commerce platforms to optimize streamer strategies and guiding the anthropomorphic development of AI streamers.

## Introduction

Live e-commerce, as an emerging business model resulting from the deep integration of the e-commerce and live-streaming industries, is driving a new wave of transformation in the consumer market through its high interactivity and strong entertainment value [1,2]. As mobile networks mature and applications continue to improve, live e-commerce significantly boosts consumer engagement and purchase conversion rates. This is achieved through mechanisms such as real-time Q&A, product demonstrations, and time-limited promotions. During the COVID-19 pandemic, global live shopping activity increased by 76% year-on-year [3], with the Chinese market standing out. The market size in China expanded from 420 billion RMB in 2019 to nearly 5 trillion RMB in 2023, and it is projected to exceed 8.16 trillion RMB by 2026 [4].

Despite the rapid growth of live e-commerce, a research gap remains in academia concerning the role of streamers, who are a core element of the live-streaming ecosystem. In particular, there is limited understanding of how differences between AI streamers and human streamers influence consumer purchase intention. Existing studies primarily focus on the independent influence of either human streamers or AI streamers, lacking a systematic exploration of the differences between the two. Research on human streamers mainly explores how their characteristics affect consumer purchase intention. These characteristics include streamer expertise [5], voice characteristics [6], responsiveness, popularity, and ethical reputation [7]. In addition, some studies emphasize the impact of human streamers’ self-presentation behaviors [8] and role performance [9] on consumer behavior.

On the other hand, AI streamers are gaining attention due to their automation and cost efficiency, yet their limited emotional expression capabilities may introduce complexities in consumer decision-making [10,11]. While these studies provide valuable insights into the impact of streamers on consumer behavior, they fall short of systematically exploring the mechanisms through which the differences between AI streamers and human streamers influence consumer purchase intention in live e-commerce.

To address this research gap, this study adopts grounded theory, a methodology developed by [12], to investigate the mechanisms by which differences between AI and human streamers influence consumer purchase intention. Grounded theory is an inductive, bottom-up research approach that emphasizes theory generation directly from data rather than testing pre-existing hypotheses. Unlike traditional top-down empirical research, this method begins with raw data [13]. It systematically extracts concepts and categories through open coding, axial coding, and selective coding. These elements are then used to construct theoretical models. This iterative process involves refining and categorizing concepts until theoretical saturation is reached, ensuring that no new categories emerge. This study analyzes consumers’ historical live shopping experiences to identify key factors through which AI and human streamers influence purchase intention. Based on these findings, it constructs a theoretical model to reveal the underlying mechanisms in live e-commerce.

This study investigates how differences between AI and human streamers affect consumer purchase intention in live e-commerce. Theoretically, this study offers a novel framework for understanding consumer behavior. It systematically compares the impact of differences between AI and human streamers, addressing a gap in prior research that has focused on only one type of streamer. Practically, the findings offer actionable insights for optimizing streamer strategies. They help e-commerce platforms leverage the strengths of AI streamers to enhance consumer engagement and promote business growth.

## Literature review

### Live-streaming purchase intention

Live e-commerce, as an emerging business model combining real-time interaction and online shopping, has profoundly transformed consumer shopping experiences and the operational models of the retail industry [14]. Through immersive environments and instant communication, live e-commerce not only enhances consumer engagement but also significantly increases brand loyalty and purchase intention. Existing studies indicate that understanding the mechanisms behind consumer purchase intention in the live e-commerce context is crucial for optimizing corporate marketing strategies and improving user experiences [15]. A review of the existing literature reveals that consumer purchase intention is primarily influenced by technological, social, and psychological factors.

First, from the perspective of technological factors, the system quality, real-time interaction features, and immersive visual effects of live e-commerce significantly enhance consumer trust while reducing perceived risks, thereby increasing purchase intention [16]. For example, high-quality live streaming and smooth interactive functions allow consumers to understand product details in real time, while instant feedback alleviates concerns and strengthens confidence in the product. Moreover, technological innovations offer an entertainment-driven shopping experience, enabling consumers to make purchase decisions more easily in a relaxed and enjoyable atmosphere [17].

Second, social factors play a critical role in shaping purchase intention in live e-commerce. Studies have shown that the social presence and credibility of streamers are key drivers of consumer decision-making [18]. High-influence streamers foster a virtual community atmosphere, enhancing consumer trust and engagement [19]. Additionally, interactions among viewers and the social environment further encourage the formation of consumer purchase intentions. This social dimension not only stimulates consumer interest in live content but also triggers impulsive buying behavior [20].

Third, from the psychological perspective, emotional engagement, perceived enjoyment, and trust are core elements that shape purchase intention. Positive emotional interactions in live-streaming environments such as warmth, authenticity, and perceived intimacy can foster consumer involvement and strengthen their intention to purchase. Similarly, recent empirical evidence confirms that influencer trust and attachment are critical psychological mechanisms that drive consumer purchase intention in live-streaming contexts, with attachment exerting a stronger effect than trust [21].

Despite the significant progress in understanding technological, social, and psychological dimensions, several research gaps remain. First, most studies focus on single countries or regions, lacking systematic analysis of cross-cultural consumer behaviors. Second, existing research predominantly employs cross-sectional designs, which fail to capture how consumer behavior evolves over time [22]. Third, the relative influence of streamers’ personal attributes and technological features on consumer behavior has not been thoroughly compared. These limitations constrain a comprehensive understanding of the mechanisms that underlie purchase intention in live e-commerce.

Given these gaps, this study aims to explore the interactive effects of technological and social factors on consumer purchase intention. By addressing these issues, the study seeks to bridge the research gap, contribute to theoretical development, and provide practical insights for businesses seeking to enhance their live-streaming strategies.

### AI streamer

In recent years, the rapid advancement of artificial intelligence (AI) technology has significantly transformed the e-commerce landscape, particularly with the emergence of AI streamers, which have injected new vitality into live e-commerce. As virtual entities developed using AI technologies, AI streamers simulate human-like interaction and emotional expression through natural language processing (NLP), computer vision, and affective computing [23]. This innovative format not only enhances marketing efficiency but also generates substantial commercial value by maintaining a consistent brand image and providing precise, personalized recommendations [24]. However, compared to human streamers, AI streamers face limitations in emotional authenticity and real-time responsiveness, which hinder their ability to build relationships and maintain interaction quality with consumers [25]. While research suggests that AI-driven technology can optimize the user experience, there remains a lack of systematic exploration regarding how AI streamers influence consumer trust, purchase intention, and the realization of commercial value [26].

Existing research on AI streamers primarily focuses on three areas: technical characteristics, consumer trust and acceptance, and mechanisms influencing purchase behavior.

First, regarding technical characteristics, studies explore how AI streamers use technologies such as voice interaction, affective generation, and personalized recommendation to achieve virtual interaction [27]. For instance, AI streamers, powered by machine learning algorithms, can accurately predict consumer preferences and significantly improve conversion rates. However, these studies primarily emphasize the technological implementation while overlooking the emotional connection between technology and consumer experience.

Second, consumer trust and acceptance is a central focus of AI streamer research. Studies indicate that anthropomorphic design and high-quality interaction are critical factors in increasing consumer acceptance and trust [23]. AI streamers, through lifelike facial expressions and voice imitation, enhance emotional bonding with consumers. However, consumer acceptance is highly context-dependent: in markets with moderate information asymmetry, firms can strategically combine pricing and AI streamer acceptance levels to signal product quality, whereas high asymmetry forces firms to incur additional costs to establish credibility [28]. This suggests that AI streamers’ effectiveness is not merely a function of technical design but also of firms’ ability to adapt signaling strategies to market conditions. Further investigation is needed into how trust evolves over time and the mechanisms for maintaining long-term consumer engagement [25].

Third, the mechanisms influencing purchase behavior are gaining increasing attention. Research suggests that AI streamers, through highly personalized recommendations and logically coherent communication, reduce consumers’ cognitive burden, thereby enhancing purchase intention [24]. However, studies on human streamers indicate that streamer attachment has a stronger impact on purchase intention than streamer trust, with platform-specific variations in effectiveness [29]. This highlights a key limitation of AI streamers: their current inability to replicate the depth of emotional attachment that human streamers cultivate, particularly in impulse-driven consumption contexts [26].

Although existing studies have made progress in understanding the technical and behavioral aspects of AI streamers, there remains a lack of systematic exploration of the complex relationship between AI streamers’ technical characteristics, consumer trust, and purchase behavior. Therefore, this study focuses on how the technical characteristics of AI streamers influence consumer purchase intention through consumer trust, particularly examining whether this relationship changes significantly in highly anthropomorphic virtual environments.

In summary, AI streamers, as an innovative integration of AI technology and e-commerce, present vast application potential. This study will focus on how highly anthropomorphic AI streamers influence consumer trust and purchase intention through the interplay of technology and emotional interaction, addressing a critical research gap and providing insights for both theoretical development and practical implementation in live e-commerce.

## Research design and data collection

### Research method

Grounded theory is well-suited for exploring micro-level, action-oriented, and process-based research questions. It emphasizes collecting data from real-life contexts in order to analyze the psychological activities and meaning-construction processes of research subjects. Compared to web-scraped cross-sectional data, grounded theory enables a more comprehensive and in-depth exploration of various aspects of consumer engagement and purchase behavior in live e-commerce [30]. In this study, the interview process followed the grounded theory methodology, guiding consumers to share their authentic experiences while watching both AI streamers and human streamers. This approach yielded valuable first-hand data that reflect the real-time emotional and cognitive responses of consumers. The interview data were then systematically processed using open coding, axial coding, and selective coding [31]. This process involved refining concepts, categories, and relational connotations while continuously theoretically sampling to enrich the data until theoretical saturation was achieved. Through this iterative process, the study constructs a theoretical model that reveals the internal mechanisms through which AI streamers and human streamers differently influence consumer purchase intention in live e-commerce settings. This model aims to uncover the deeper reasons behind these differences. It also provides theoretical insights into the consumer decision-making process.

### Interview outline design

The interview outline adopted a problem-oriented approach, combining inductive and deductive reasoning to gather first-hand information. Based on a literature review and real-world e-commerce issues, the outline was designed by one e-commerce professor and three doctoral students. It consists of three sections: (1) Basic information about the participants, (2) Recollection of past live-streaming shopping experiences, and (3) Perceptions of AI and human streamers and factors influencing purchase intention. A pilot interview was conducted with six experienced consumers, and the outline was refined based on their feedback, leading to the final interview outline, as shown in Table 1.

**Table 1:** Interview outline.

No.	Question Overview
1	Do you have experience watching live-streaming e-commerce? How often do you watch it?
2	Do you prefer watching live streams hosted by AI streamers or human streamers? Why?
3	Have you ever purchased products from a live stream hosted by an AI or human streamer? Please describe your purchasing experience.
4	What are the main differences between AI and human streamers in terms of appearance, voice, facial expressions, and dynamic performance? Do these factors affect your viewing experience?
5	What do you think are the advantages and disadvantages of AI streamers compared to human streamers regarding content delivery, interaction style, and emotional expression?
6	In product presentation and recommendation, which do you find more persuasive, AI streamers or human streamers? Why?
7	During a live stream, do you prefer interacting with AI streamers or human streamers? Why?
8	Do you think watching live streams hosted by AI or human streamers influences your purchase intention? If yes, what are the main influencing factors?
9	If AI streamers are further improved in the future (e.g., enhanced interaction and emotional expression), would you be more willing to accept them? Why?
10	What are your views on the future development trends of AI and human streamers in live-streaming e-commerce?

### Selection of research subjects and data collection

Interview data are the preferred method for qualitative research as they authentically reflect the thoughts and concepts underlying social phenomena and summarize the theoretical framework through relationships between categories [32]. This study adopts a qualitative research approach to deepen the understanding of how the differences between AI streamers and human streamers affect consumer purchase intention. Purposeful sampling was employed to select a specific group aligned with the research objectives.

According to reports from the China Consumers Association [33] and Sina Weibo Hotspot [34], individuals born between the 1980s and 2000s are the primary participants in live-streaming e-commerce, with 20–30-year-olds being the most active, accounting for over 55% of users. This age group has higher acceptance of new technologies and greater proficiency in using digital media, making them central participants in live-streaming e-commerce. As early adopters of emerging media, they offer valuable insights into their perceptions and experiences with live-streaming and AI streamers, which are essential for this study.

Based on the criteria, the study follows the typical sampling principle, focusing on young and middle-aged consumers who have experience with live-streaming on e-commerce platforms. This age group was selected due to their higher sensitivity to emerging technologies. Participants, aged 18 and above, were recruited from a university community, particularly from disciplines related to e-commerce and consumer behavior. This group’s independent thinking, decision-making abilities, and availability for in-depth interviews make them ideal candidates for this study. Furthermore, their academic background closely aligns with the research theme, ensuring that they can provide professional insights and comprehensive perspectives on live-streaming e-commerce, which is crucial for understanding consumer behavior in this context.

After defining the interview framework and target participants, we conducted interviews over a two-week period, collecting 16 hours of audio recordings. The recordings were transcribed and manually verified, resulting in 28 documents totaling approximately 35,000 words. Two respondents were interviewed daily, and after each session, the questions were reviewed and adjusted to optimize subsequent interviews and ensure the collection of relevant research content.

Following the recommendations of Holstein and Gubrium (2001) [35], who suggest that a sample size of 28–40 is ideal for in-depth interviews to ensure validity and focus, we conducted 28 face-to-face interviews between November 10 and 25, 2024. The sample included 16 women and 12 men, with each interview lasting between 60 and 90 minutes. The basic information of the respondents is presented in Table 2.

**Table 2:** Basic information of interviewees.

Item	Category	Samples(n = 28)	Percentage(%)
Gender	Male	12	42.86%
	Female	16	57.14%
Age	20-30	19	67.86%
	30-40	7	25.00%
	40-50	2	7.14%
Education	Undergraduate	6	21.43%
	Master	13	46.43%
	Doctor	9	32.14%
Experience on online shopping	Less than 3 years	2	7.14%
	3-5 years	5	17.86%
	More than 5 years	21	75.00%

Following theoretical saturation principles, we randomly sampled the interview records and divided them into two subsets. Specifically, two-thirds of the in-depth interview data were used for coding to extract concepts and categories, while the remaining samples were reserved for saturation testing to verify whether new categories emerge. The coding was carried out until no additional categories or concepts emerged from the remaining interview data, confirming that theoretical saturation had been achieved. 22 interview transcripts were imported into NVivo 11 for coding and analysis.

### Data analysis

This study adopts the qualitative analysis method proposed by Strauss and Corbin [36], employing a three-stage coding process: open coding, axial coding, and selective coding. Open coding is used to identify key concepts, axial coding establishes the data framework, and selective coding extracts the core themes.

### Open coding

Open coding involves a line-by-line analysis, comparison, and categorization of raw data to extract the core viewpoints of participants through conceptualization and categorization [31]. The primary goal is to transform participants’ language into abstract concepts and general categories. To minimize subjective bias during the coding process, concept identification and category classification were conducted collectively by the research team, with consensus reached through team discussions and expert consultations.

The study uses NVivo qualitative analysis software to aid in the coding process. A total of 22 interview transcripts were imported into the software and then coded and analyzed. During the process, researchers conducted a word-by-word analysis to identify keywords. These keywords were labeled based on their semantic meaning. Labels that appeared three or more times were categorized and refined to form initial categories, which ultimately led to the development of open coding. Throughout the process, researchers prioritized using participants’ exact words to minimize bias and accurately reflect their intentions [37].

Upon completing the open coding stage, 29 concepts, 16 initial categories (e.g., Appearance and Image, Sound Quality), and 15 subcategories were identified. As shown in Table 3, each subcategory is clearly linked to its initial category, corresponding concept, and representative original statements from the interviews.

**Table 3:** Results of conceptualization and categorization of open coding.

Subcategories	Initial Categories	Concepts	Representative Original Statements
B1 Appearance and Image	A1 Sensory Attraction	aa1 Degree of Anthropomorphism	AI streamers have a high degree of anthropomorphism and look so realistic that it’s hard to tell they are not human.
		aa2 Attractiveness	Human streamers are generally more attractive, which makes them more appealing to watch.
B2 Sound Quality	A2 Speech Style	aa3 Standardized Speech	AI streamers’ speech is too standardized and smooth, making it sound flat and lacking in warmth.
		aa4 Expressiveness	Human streamers’ speech is full of emotional ups and downs, making their delivery more passionate and engaging.
B3 Dynamic Performance	A3 Action and Expression	aa5 Mechanical Movements and Expressions	AI streamers’ gestures and expressions are rigid and repetitive. While precise, they lack the flexible reactions of humans.
		aa6 Flexible Movements and Expressions	Human streamers’ body language and facial expressions are dynamic and engaging, especially during exciting moments.
B4 Service Capability	A4 Program Effectiveness	aa7 Lack of Flexibility	AI streamers are rigid and only present product information without much improvisation.
		aa8 Strong Program Effectiveness	Human streamers create entertaining content, sometimes using humor or impromptu skits to explain products.
B5 Knowledge Reserve	A5 Product Knowledge	aa9 Repetitive Information	AI streamers simply repeat pre-set scripts and cannot link product details to current trends or real-life examples.
		aa10 Rich Social Knowledge	Human streamers not only remember product details but also connect them with social trends or personal anecdotes.
B6 Spoken Pronunciation	A6 Pronunciation Standard	aa11 Fluent and Accurate	AI streamers’ speech is always smooth and accurate in pronunciation.
		aa12 Non-standard Pronunciation	Human streamers’ speech varies, and some have regional accents or unclear articulation.
B7 Character Setting	A7 Personalization	aa13 Reused Appearance	AI streamers’ appearance is often reused across multiple live streams, leading to aesthetic fatigue and a lack of uniqueness.
		aa14 Personalized Image	Human streamers have distinct identities and personalities, making some particularly captivating and memorable.
B8 Emotional Disclosure	A8 Emotional Expression	aa15 Lack of Emotional Variability	AI streamers maintain a constant smile without emotional fluctuation, which can feel eerie after prolonged viewing.
		aa16 Emotional Intensity	Human streamers exhibit emotional highs and lows, especially when urging purchases, creating a stronger emotional connection.
B9 Accompany and Interact	A9 Interaction Timeliness	aa17 Response Delay	AI streamers often delay or fail to respond to audience questions promptly.
		aa18 Timely Interaction	Human streamers promptly respond to comments and greet viewers by their IDs, creating a sense of personal attention.
B10 Content Novelty	A10 Content Innovation	aa19 Lack of Novelty	AI streamers’ content feels mechanical and repetitive, lacking the element of surprise.
		aa20 Innovative Content	Human streamers introduce new topics or personal opinions, making their content feel fresh and engaging.
B11 Interactive Enjoyment	A11 Interactive Flexibility	aa21 Monotonous Interaction	Although AI streamers can respond to simple queries, their interaction feels scripted and lacks spontaneity.
		aa22 Engaging Interaction	Human streamers adapt to audience feedback, play games, and conduct interactive Q&As, making the session lively and fun.
B12 Emotional Authenticity	A12 Emotional Resonance	aa23 Emotional Coldness	AI streamers feel like cold machines, while their tone is polished, their emotional delivery lacks genuine warmth.
		aa24 Emotional Resonance	Human streamers fully express their emotions, when they love a product, their joy and excitement are contagious.
B13 Personalization Fit	A13 Personalized Recommendation	aa25 Fixed Content	AI streamers follow fixed scripts regardless of audience preferences, lacking the ability to customize product suggestions.
		aa26 Flexible Adaptation	Human streamers adjust their content based on audience comments, offering tailored recommendations and insider tips.
B14 Consumer Purpose	A14 Purchase Motivation	aa27 Influence on Purchase Intention	If I’m familiar with a product, I’m fine buying from an AI streamer. For unfamiliar products, I prefer human streamers due to trust concerns.
	A15 Viewing Motivation	aa28 Viewing Intent	If I’m watching for entertainment, I likely won’t buy. But if I’m already planning to buy something, I am more likely to purchase.
B15 Purchase Intention	A16 Purchase Decision	aa29 Purchase Decision Driver	Emotional enthusiasm from human streamers often persuades me to buy products. AI streamers’ clear presentation is also convincing.

### Axial coding

Axial coding connects independent categories from open coding, revealing hierarchical and logical relationships to develop main categories [36]. Compared to open coding, it offers greater explanatory power by systematically clustering categories through causal, functional, and structural relationships [37]. Using this method, we linked categories through causal relationships and organized them into main categories, as shown in Table 4. This process provides a comprehensive framework for understanding the impact of AI and human streamers on consumer purchase intention.

**Table 4:** Axial coding results and relationship connotation.

Main Categories	Subcategories	Relational Connotations
Sensory Difference	Appearance and Image	Differences between AI and human streamers in terms of appearance, makeup, and outfit styling.
	Sound Quality	Differences between AI and human streamers in tone, voice timbre, and sound quality.
	Dynamic Performance	Differences between AI and human streamers in body movements and facial expressions.
Functional Difference	Spoken Pronunciation	Differences between AI and human streamers in speech fluency and pronunciation accuracy.
	Service Capability	Differences between AI and human streamers in improvisation and conversational communication.
	Knowledge Reserve	Differences between AI and human streamers in knowledge association and awareness of current events.
Affective Difference	Character Setting	Differences between AI and human streamers in identity, personality traits, and emotional representation.
	Emotional Disclosure	Differences between AI and human streamers in emotional expression and emotional delivery.
	Accompany and Interact	Differences between AI and human streamers in real-time emotional engagement and interaction quality.
Perceived Entertainment	Content Novelty	Consumers’ perception of AI and human streamers’ ability to introduce creative content and new topics.
	Interactive Enjoyment	Consumers’ perception of AI and human streamers’ interaction style, responsiveness, and entertainment value.
Social Presence	Emotional Authenticity	Consumers’ perception of the authenticity of emotional expression and emotional variability between AI and human streamers.
	Personalization Fit	Consumers’ perception of AI and human streamers’ ability to provide personalized recommendations and responsive interaction.
Consumer Purpose	Purchase Motivation	Consumers’ intention and motivation to make a purchase while watching live streams.
	Viewing Motivation	Consumers’ primary purpose for watching live streams.
Purchase Intention	Purchase Decision	Consumers’ purchase motivation based on their perceived trust in the sensory, functional, and affective differences between AI and human streamers.

### Selective coding

**Fig 1:**
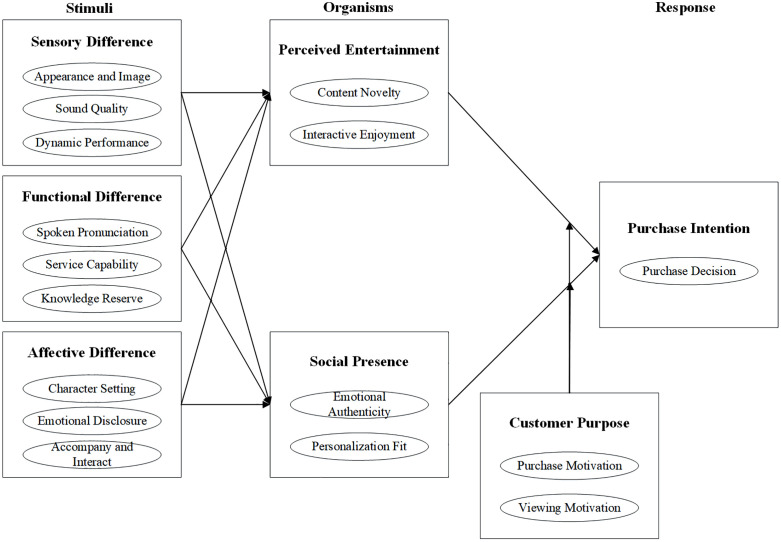
Relationship of main categories.

Selective coding further analyzes the relationships among these categories, aiming to identify a core category through an integrated storyline. After in-depth discussions, the research team identified purchase intention as the core category. Based on this, a research model was developed, as illustrated in Fig 1. This model includes sensory difference, affective difference, and functional difference, affective difference, and functional difference as stimulus factiors, perceived entertainment and social presence as organism factors, and purchase intention as the response factors, with consumer purpose serving as a moderating factor. The model explains how differences between AI streamers and human streamers in terms of sensory, affective, and functional dimensions affect purchase intention through the mediating effects of perceived entertainment and social presence, with consumer purpose moderating these effects.

### Theoretical saturation test

To validate the model’s accuracy, the remaining one-third of the interview data were subjected to the same coding and analysis process for theoretical saturation testing. No new categories or concepts emerged. This indicates that the proposed concepts and categories comprehensively capture the mechanisms behind the differential effects of AI and human streamers on purchase intention, thus achieving theoretical saturation.

### Post-hoc analysis

Given the central role of trust in shaping purchase intention in e-commerce, we conducted a post-hoc analysis to further explore this dimension. While the original interview data did not explicitly capture trust-related categories, we carried out an additional follow-up with the same group of participants. Three specific questions were raised: (1) Do you trust AI streamers? (2) Does the advancement of AI technology influence your trust in streamers? (3) Does your trust in AI streamers affect your purchase intention?

The results indicate several consistent patterns. First, the majority of respondents (approximately 93%) reported that they generally trust AI streamers. Their main reason was that AI streamers typically follow predetermined scripts and are less likely to engage in exaggerated claims or deceptive marketing compared to some human streamers. This standardized communication style was perceived as reliable, even if somewhat mechanical. Second, most participants noted that technological progress did not directly increase or decrease their trust in AI streamers. Instead, they emphasized that improvements in AI blurred the boundary between AI and human streamers, making it harder to distinguish between the two. Finally, participants overwhelmingly stated that their trust in AI streamers had little direct impact on their purchase intention. Instead, they stressed that product quality, live-streaming atmosphere, and interactive engagement were more decisive in driving their buying behavior.

These findings suggest that while AI streamers are generally perceived as trustworthy, trust alone is not a critical factor influencing consumer purchase intention. Rather, trust functions as a baseline condition, whereas emotional connection, entertainment, and social presence serve as stronger motivators.

### Model explanation

Based on grounded theory, this study identifies the key differentiating factors between AI and human streamers, including sensory difference, affective difference, and functional difference, and their mediating effects on consumer purchase intention through perceived entertainment and social presence. Using the stimulus-organism-response (S-O-R) model as a theoretical foundation, the research framework conceptualizes AI and human streamer differences (including sensory, affective, and functional differences) as stimulus factors, with perceived entertainment and social presence as organism factors, and purchase intention as the response factor.

### Analysis of sensory differences affecting perceived entertainment and social presence

In the live e-commerce context, sensory difference refers to variations in external perceptible cues that shape consumers’ sensory pleasure and sense of immersion. These sensory cues influence purchase intention primarily by enhancing perceived entertainment. When the visual presentation is attractive, the voice expressive, and the body movements natural, viewers experience stronger enjoyment and immersion, which in turn fosters a more positive attitude toward the product and a greater willingness to buy. Compared with AI streamers, whose appearance and speech are often standardized and whose gestures remain mechanically repetitive, human streamers exhibit more dynamic and emotionally expressive sensory performances. Their personalized appearance, varied vocal tones, and flexible gestures not only make the viewing process more entertaining but also create a vivid social atmosphere that strengthens consumer engagement and ultimately increases purchase intention. Within the sensory difference category, we identified three key dimensions: appearance and image, sound quality, and dynamic performance.

Appearance and image refer to the streamer’s physical appearance, style, and overall visual presentation during live streaming. Research by Guo et al. [38] indicates that a streamer’s aesthetic appeal positively affects consumer viewing and purchase intentions by delivering both hedonic and utilitarian value. Although AI streamers are highly anthropomorphized, their standardized and artificial nature limits their ability to evoke emotional resonance and provide rich entertainment experiences. They maintain a clean and consistent appearance. However, they lack the unique charm and emotional variability of human streamers, which reduces the sense of immersion and interactivity for viewers. In contrast, human streamers present personalized and diverse visual styles tailored to different scenarios. Their natural appearance conveys genuine emotions, strengthening perceived entertainment and social presence. This ability to create an authentic social atmosphere fosters immersion and actively encourages viewer participation.

Sound quality encompasses the tone, pitch, speech rate, rhythm, and emotional expression of a streamer’s voice. Previous research suggests that a higher pitch is often associated with positive emotions such as happiness, enthusiasm, and friendliness [39], which can trigger emotional contagion, allowing a streamer’s emotions to be transferred to the audience [6]. While AI streamers deliver clear and accurate speech, their mechanical and over-standardized delivery lacks emotional variability and personal expression, making it difficult for viewers to experience emotional resonance and engagement. This deficiency weakens their ability to foster social presence and entertainment experiences. In contrast, human streamers exhibit emotional expressiveness, dynamically adjusting their tone and emotional delivery to match the content. Through emotional fluctuations and variations in speech rhythm, human streamers build stronger emotional connections with their audience. This enhances perceived entertainment and social presence, creating a more immersive social atmosphere.

Dynamic performance refers to the streamer’s interactive capabilities through body language, facial expressions, and physical gestures, which are critical to shaping perceived entertainment and social presence. AI streamers often display fixed, programmed movements and facial expressions, lacking the spontaneity and flexibility of human reactions. This rigidity makes it difficult for viewers to feel engaged. It can also create a sense of distance and artificiality, diminishing both entertainment value and social connection. In contrast, human streamers offer natural and varied dynamic performances, using eye contact, smiles, gestures, and other forms of body language to convey emotions effectively. This flexible expressiveness fosters genuine emotional exchanges, enhancing entertainment experiences and social participation. Such interactive performances allow viewers to become more immersed in the social interaction environment, significantly improving both perceived entertainment and social presence.

### Analysis of functional differences affecting perceived entertainment and social presence

In live e-commerce, functional difference concerns how streamers deliver information and provide service, including spoken pronunciation, service capability, and knowledge reserve. These functional elements influence purchase intention by shaping consumers’ cognitive evaluation and decision-making efficiency. Clear pronunciation, timely responses, and relevant product knowledge reduce uncertainty, build trust, and make it easier for consumers to form purchase decisions. AI streamers typically excel in standardized delivery and consistent product descriptions, which satisfy viewers seeking accuracy and efficiency. However, they often lack flexibility and improvisation in addressing unexpected questions or tailoring content. Human streamers, in contrast, can adjust their speech patterns, incorporate humor, and draw on broader knowledge to connect products with current trends or personal experiences. This adaptability not only improves the informational value of the stream but also enhances engagement, thereby fostering stronger purchase intention.

Spoken pronunciation refers to how streamers communicate with audiences, including the accuracy of pronunciation and linguistic habits. AI streamers deliver speech that is standardized and fluent. However, their lack of emotional variation and personalized expression makes their communication seem mechanical and monotonous, reducing the audience’s enjoyment and emotional resonance. This diminishes their ability to enhance perceived entertainment and social participation. In contrast, human streamers adopt a more emotional and flexible approach, adjusting their speech rate, intonation, and tone based on the situation. Through dynamic vocal expression, human streamers convey passion and sincerity. This strengthens the audience’s immersion and entertainment experience, while fostering emotional bonds and enhancing social presence.

Service capability reflects the streamer’s professional knowledge, on-the-spot responsiveness, and ability to engage with the audience. In live e-commerce, audiences frequently ask streamers questions to clarify product details [40]. AI streamers, limited by pre-programmed scripts, can deliver accurate product information but lack flexibility and adaptability to real-time feedback, which reduces their ability to sustain perceived entertainment and social presence. Conversely, human streamers excel in improvisation and interaction. They can address audience concerns in real time and integrate humor, performance elements, and engagement strategies into their presentations. This responsiveness enhances entertainment value, strengthens emotional connections, and fosters a more immersive and participatory environment, increasing social presence.

Knowledge reserve refers to the streamer’s ability to present expertise, industry insights, and current social trends. In live e-commerce, streamers serve as key marketing agents [41], and their professional competence, shaped by their knowledge, experience, and achievements, is crucial to influencing consumer behavior [38]. Although AI streamers can deliver accurate and consistent information, they lack sensitivity to real-time social trends and personalized storytelling. This restricts their ability to engage in complex discussions or foster emotional connections, weakening both perceived entertainment and social presence. In contrast, human streamers can contextualize their product presentations by integrating personal experiences and current events, making their delivery more engaging and authentic. This narrative richness enhances the audience’s entertainment experience, strengthens emotional engagement, and increases social presence within the live-streaming environment.

### Analysis of affective differences affecting perceived entertainment and social presence

Affective difference refers to disparities in emotional expression, personality traits, and interactive style between streamers. These affective qualities shape how viewers experience social presence and emotional resonance, which are critical drivers of purchase intention. When streamers display genuine emotions, respond warmly, and create a sense of companionship, audiences feel more connected and immersed. This emotional bond enhances trust and makes consumers more willing to act on purchase impulses. AI streamers, while capable of simulating basic emotions, usually present scripted and uniform expressions that limit their ability to convey authenticity or spontaneity. Human streamers, by contrast, reveal natural fluctuations in mood, personalize interactions, and share personal stories that strengthen the sense of intimacy. Their authentic emotional disclosure and adaptive engagement deepen social presence, encourage stronger consumer involvement, and ultimately raise purchase intention. The affective difference is primarily reflected in three dimensions: character setting, emotional disclosure, and accompany and interact.

Character setting refers to the streamer’s image, identity, and personality traits. These characteristics form the foundation for emotional expression and influence the audience’s sense of identification and emotional resonance. AI streamers rely on pre-set virtual personas with a degree of anthropomorphism. However, their fixed and standardized image lacks individuality and emotional depth, making it difficult to convey complex emotions or establish emotional bonds with viewers. This limitation weakens entertainment experiences and social interaction. In contrast, human streamers draw on real-life experiences and genuine emotions, which make their identities and personalities more diverse and authentic. By sharing personal stories and emotional experiences, human streamers reduce the emotional distance between themselves and their audience, enhancing emotional resonance and significantly improving perceived entertainment, social presence, and audience participation.

Emotional disclosure refers to the streamer’s ability to express emotional fluctuations and attitudes during live streaming, which is a crucial factor in fostering emotional resonance and engagement. AI streamers, limited by pre-programmed emotional scripts, can simulate basic emotions such as joy but lack dynamic emotional responses and individualized reactions. This mechanical and inauthentic emotional display makes it difficult for viewers to feel connected, thereby diminishing both entertainment value and social presence. In contrast, human streamers exhibit spontaneous and context-sensitive emotional responses, such as joy, surprise, or excitement, which they adjust in real-time based on different scenarios. These genuine emotional expressions not only strengthen perceived entertainment but also deepen social presence, allowing viewers to feel actively involved in real-time social interactions and enhancing their overall sense of participation.

Accompany and interact refers to real-time emotional communication between the streamer and the audience. Interactive ability reflects how well a streamer responds to consumer feedback [7], and higher interaction quality increases consumer enjoyment [42]. AI streamers, constrained by programmed responses, provide rigid and standardized interactions. Although they can respond to questions through text or voice, their responses often lack flexibility and emotional warmth, reducing both entertainment experiences and social presence. In contrast, human streamers deliver emotional and adaptive responses, tailoring their communication based on audience feedback. This emotional attentiveness is further enriched by their ability to inject humor and warmth. This makes viewers feel valued and enhances their sense of belonging. Such emotion-driven interactions significantly improve the audience’s engagement, social presence, and overall investment in the live-streaming experience.

### Moderating role of consumer purpose

Consumer purpose refers to the underlying motives and goals that drive consumer behavior in live-streaming e-commerce. It moderates how sensory, functional, and affective differences influence perceived entertainment, social presence, and ultimately purchase intention. When the purpose is purchase-oriented, consumers rely more on systematic information processing, making functional differences such as clarity, accuracy, and efficiency especially important. In this context, AI streamers are often favored for their standardized delivery and consistent product knowledge. In contrast, when the purpose is viewing-oriented, consumers focus on affective and sensory experiences that enhance enjoyment and emotional connection. Human streamers, with their expressive emotions, dynamic performances, and interactive engagement, provide stronger entertainment value and social presence, which in turn strengthen consumer involvement and purchase intention.

Sensory difference, particularly appearance and image, sound quality, and dynamic performance, is a key factor influencing perceived entertainment, and consumer purpose significantly moderates this relationship. When purchase motivation is high, consumers tend to be more receptive to standardized and simplified sensory cues. For instance, when efficiency is the primary goal, AI streamers, with their standardized appearance and clear sound delivery, better meet the needs of consumers seeking a quick purchasing process. In contrast, when entertainment motivation is stronger, consumers value personalized and emotionally expressive sensory stimuli. In these cases, human streamers, with their dynamic expressions and emotional richness, offer a more engaging and entertaining experience. Thus, efficiency-oriented consumers may prefer AI streamers for their concise and consistent presentation, whereas entertainment-driven consumers are more inclined to choose human streamers for their emotional expressiveness and interactivity.

When consumers are primarily motivated by entertainment, they are more attracted to the streamer’s emotional expression, particularly human streamers who offer emotional disclosure and accompanying interaction, which enhances social presence. Many interviewees indicated that when they watch live streams for entertainment, the emotional dynamics and personal engagement from human streamers create a deeper sense of immersion, increasing their participation and social belonging. However, when purchase motivation dominates, the significance of affective differences diminishes. For AI streamers, their standardized emotional delivery and mechanical consistency align with consumers’ desire for efficient information processing. In such scenarios, consumers prioritize information accuracy and delivery efficiency over emotional warmth. Although AI streamers lack emotional depth, they still offer sufficient social presence to support decision-making. This allows efficiency-oriented consumers to focus on product details rather than the emotional aspects of the interaction.

The moderating effect of consumer purpose on functional difference is primarily reflected in information needs. When consumers have a strong purchase intent, they prioritize the accuracy of the streamer’s service capability and knowledge reserve. In this context, AI streamers, with their precise pronunciation and standardized product knowledge, effectively meet these needs, enhancing trust in the content and social presence. Conversely, for entertainment-driven consumers, the impact of functional difference is more nuanced. Although service capability remains important, interactivity and entertainment value become equally crucial. Human streamers, with their flexible speech, improvisational skills, and broad social knowledge, not only provide accurate information but also enrich the viewing experience with humor and engagement. Entertainment-oriented consumers tend to prefer human streamers, as their personalized communication and dynamic performance enhance both perceived entertainment and social presence, rendering the live-streaming experience more engaging and immersive.

## Discussion

This study explores how differences between AI streamers and human streamers in live e-commerce influence consumer purchase intention, revealing that sensory, functional, and affective differences shape perceived entertainment and social presence, which in turn drive purchase decisions. Sensory differences, such as appearance and sound, appeal to efficiency-driven consumers (AI streamers) or entertainment-focused viewers (human streamers). Functional differences highlight AI streamers’ precision in information delivery versus human streamers’ improvisational skills and emotional engagement. Affective differences emphasize genuine emotional expression and interaction quality, where human streamers excel in fostering social presence and emotional resonance. Consumer purpose moderates these effects, prioritizing efficiency for purchase intent or emotional engagement for entertainment.

Building on these findings, the study offers actionable implications for stream selection strategies in live e-commerce. Specifically, AI streamers are more suitable for utilitarian, standardized products such as consumer electronics, office supplies, or commodity goods, where information clarity, consistency, and speed are prioritized. These products often attract goal-oriented consumers who value streamlined communication and efficient decision-making. In contrast, human streamers are more effective for experience-based or emotionally engaging products such as cosmetics, fashion, food, and luxury items. These product categories benefit from emotional storytelling, nuanced interaction, and a strong sense of presence—all of which human streamers are better equipped to deliver.

Moreover, the choice between AI and human streamers may also depend on the type of merchant and their operational resources. Large-scale platforms with high-frequency product demonstrations (e.g., JD.com or logistics-oriented merchants) may benefit from AI streamers’ cost-effectiveness and consistent delivery, especially for repetitive promotional content. In contrast, boutique sellers, brand storytellers, or influencer-driven channels may prefer human streamers for building long-term customer relationships and brand equity through emotionally rich, interactive engagement.

While this study focuses on the comparative impact of AI and human streamers, our findings also suggest potential complementarities between the two. For example, a hybrid strategy could involve AI streamers presenting factual product information efficiently, followed by human streamers offering emotional resonance and real-time interaction. Such role-based coordination may maximize both cognitive clarity and emotional engagement in live-streaming environments. Although this lies beyond the current study’s scope, it offers a promising direction for future research on human–AI collaboration and task allocation in digital marketing.

Theoretically, this study advances live e-commerce research by integrating the S-O-R model with grounded theory, uncovering psychological mechanisms behind consumer behavior. Practically, it guides platforms to dynamically allocate streamer types based on product characteristics and consumer motivations, while suggesting enhancements to AI streamers’ emotional expressiveness. Limitations include sample restrictions and the qualitative nature of grounded theory, calling for future research to expand demographic diversity and adopt longitudinal or mixed-method approaches to capture evolving consumer dynamics.

## Conclusion and implications

This study, based on grounded theory, explores the mechanism by which the differences between AI streamers and human streamers influence consumer purchase intention in the context of live e-commerce. By employing a three-stage coding process, including open coding, axial coding, and selective coding, we conducted a detailed line-by-line analysis of interview data. This process resulted in the identification of seven main categories: sensory difference, affective difference, functional difference, perceived entertainment, social presence, consumer purpose, and purchase intention. Based on the S-O-R model, we developed a theoretical framework to explain how these categories interact to shape consumer purchase behavior. This research provides theoretical and practical insights by filling gaps in the literature and offering strategic guidance for e-commerce platforms and merchants. Specifically, platforms can tailor the allocation of AI and human streamers according to product categories and consumer goals—for instance, employing AI streamers for standardized products that require efficient information delivery, and human streamers for experience-driven products where emotional engagement is critical. Such actionable strategies can help platforms optimize resource allocation and enhance overall conversion rates.

### Theoretical implications

This study makes three key theoretical contributions: First, it develops a new theoretical model using grounded theory to explain how AI streamers and human streamers influence consumer purchase intention in live e-commerce. Unlike traditional survey-based methods, grounded theory enables a deep exploration of the underlying psychological mechanisms driving consumer behavior. This method is particularly effective for uncovering the emotional dynamics, interactivity, and deep psychological motivations involved in live e-commerce. Second, this study addresses a research gap by offering an empirical framework that systematically analyzes how sensory, affective, and functional differences between AI and human streamers influence perceived entertainment, social presence, and purchase intention. While conventional survey and machine-learning approaches capture surface-level trends, the layered coding process in grounded theory provides multidimensional insights into the complexity of consumer behavior. Third, by integrating the S-O-R model, this study confirms the critical role of sensory difference, affective difference, and functional difference in shaping consumer purchase decisions. This model not only enhances understanding of the interaction between technology and consumer behavior but also lays a theoretical foundation for future research on AI-human dynamics in live-streaming contexts.

### Practical implications

This study provides practical insights for e-commerce platforms, merchants, and AI developers: First, merchants should dynamically allocate streamer types based on product characteristics and consumer needs. For standardized, information-transparent products (e.g., consumer electronics or daily necessities), AI streamers are more effective due to their precise communication and efficient delivery. For experience-driven products (e.g., cosmetics or luxury goods), human streamers are preferable due to their emotional expressiveness and interactive engagement, which foster emotional resonance and purchase motivation. Second, AI developers should focus on enhancing the anthropomorphism of AI streamers by leveraging affective computing technologies. This includes embedding emotional variability into speech synthesis and simulating micro-expressions to mitigate the perception of mechanical coldness. Improved dynamic emotional capabilities could enhance consumer engagement and social presence. Third, platform managers should develop intelligent matching systems that dynamically recommend AI or human streamers based on user profiles and real-time behavioral data. Implementing a multi-dimensional evaluation framework to quantify the effectiveness of different streamer types can provide data-driven insights to optimize operational strategies. Moreover, merchants should adapt their streamer strategies based on consumer purpose. For efficiency-driven consumers, AI streamers provide concise, accurate information and facilitate quick decision-making. For entertainment-seeking audiences, human streamers create a more engaging and emotional experience. Cross-platform strategies should also align with platform characteristics: for instance, Douyin, with its entertainment-oriented user base, is better suited for human streamers, while JD.com, with a utilitarian focus, may benefit from AI-driven product demonstrations. This flexible deployment of AI and human streamers can maximize conversion rates and user retention.

### Limitations and further research

While this study provides a comprehensive model explaining the differentiated impacts of AI and human streamers on consumer purchase intention, it has several limitations: First, the sample is limited to Chinese university students and faculty, which may restrict the generalizability of the findings across different demographic groups. Future studies should expand the sample to include diverse cultural and age groups to test the universality of the proposed model. Second, the cross-sectional research design limits the ability to capture the evolution of consumer behavior over time. Given the rapid advancements in AI technology, future research could adopt longitudinal methods and mixed approaches (e.g., eye-tracking and behavioral modeling) to enhance the robustness of the findings. Third, due to methodological constraints of qualitative interviews, this study did not incorporate individual-level psychological traits such as extroversion, openness, or risk tolerance, which may influence consumers’ responses to different streamer types. Future research using quantitative or experimental designs could explore how personality traits moderate the perceived effectiveness of AI and human streamers. Additionally, the widespread adoption of AI streamers raises critical issues regarding job displacement, consumer privacy, and ethics. Future studies could investigate these socioeconomic impacts and provide policy recommendations to promote sustainable AI integration in the e-commerce ecosystem.
